# Boron Removal by Membrane Distillation: A Comparison Study

**DOI:** 10.3390/membranes10100263

**Published:** 2020-09-28

**Authors:** Abdullah Alkhudhiri, Nawaf Bin Darwish, Mohammed Wali Hakami, AbdelKader Abdullah, Ahmed Alsadun, Hosam Abu Homod

**Affiliations:** 1 National Center for Desalination & Water Treatment Technology, King Abdulaziz City for Science and Technology (KACST), Riyadh 11442, Saudi Arabia; nbindarwish@kacst.edu.sa (N.B.D.); aalsadun@kacst.edu.sa (A.A.); habuhomod@kacst.edu.sa (H.A.H.); 2 Chemical Engineering Technology Department, Jubail Industrial College, Jubail Industrial City 31961, Saudi Arabia; hakami_mw@jic.edu.sa; 3 College of Engineering, Prince Sattam bin Abdulaziz University, P.O. Box 655, AlKharj 11942, Saudi Arabia; a.abdullah@psau.edu.sa

**Keywords:** boron, membrane distillation, synthetic seawater, air gap membrane distillation, vacuum membrane distillation, permeate gap membrane distillation

## Abstract

Several Membrane Distillation (MD) technologies have been employed to remove boron from various concentrations. In this study, Vacuum Membrane Distillation (VMD), Permeate Gap Membrane Distillation (PGMD), and Air Gap Membrane Distillation (AGMD) are examined to evaluate their effectiveness when combined with several boron concentrations (1.5, 7 and 30 ppm) and operating parameters (circulation rate from 0.9 L/min to 5 L/min, feed temperature from 40 to 70 °C, and pH from 3–11). Those concentrations of boron are selected on the basis of the concentration of boron in the permeate side of the single-pass reverse osmosis (RO) system, Arabian Gulf, and contaminated brackish water. Moreover, synthetic seawater is treated to assess MD technologies’ effectiveness. A high removal efficiency of boron is accomplished by MD. AGMD, PGMD, and VMD are promising methods for the desalination industry. AGMD shows excellent boron removal, which was above 99% with a wide ranging concentration. In addition, VMD demonstrates good permeate flux compared to the other MD technologies, which were about 5.8 kg/m^2^·h for synthetic seawater. Furthermore, there is no noteworthy influence of the pH value on the boron removal efficiency.

## 1. Introduction

Boron is dispersed widely in the lithosphere and hydrosphere of the earth [1]. The biggest consumer of the boron compound (which utilizes more than fifty percent of the total world production) is the glass industry [2]. Boron is utilized in antiseptics, medical treatment, cosmetics, and the nuclear industry.

Boron concentration is strongly related to geographical location and seasonal effects [3]. For example, the average boron concentration in seawater has been reported to be about 4.5 ppm while the boron concentration level in the Arabian Gulf is about 7 ppm [3]. Commonly, boron concentration in brackish water and hot springs varies from 0.3 to 100 ppm [4].

Additionally, boron concentration in water irrigation is essential and has a major role in the quantity and quality of the crops. It should not go beyond 4 ppm, depending on the crop kind and soil properties [2].

In an aqueous environment, dissolved boron is found in several species such as mononuclear species and polynuclear boron. In a moderate pH level (seawater), Boron is mostly found as un-dissociated boric acid B(OH)_3_ or borate ion B(OH)^−^_4_ as follows [5]:

B(OH)_3_ + H_2_O ↔ B(OH)^−^_4_ + H^+^ (1)

Several factors affect boron removal via membrane technology, these are salinity, flow rate, feed pressure, feed temperature, boron concentration, and feed pH. Hilal et al. [2] pointed out that boron rejection in the permeate side decreases when the feed salinity and temperature increase. In contrast to membrane technology, Boron is almost completely removed by thermal desalination technology.

### 1.1. Membrane Technology

Reverse osmosis (RO) successfully provides a satisfying quality of freshwater with about a 98% removal of salts and other solutes in seawater. In RO desalination plants, the removal of boron from seawater is a difficult task [6]. Boron and other trace contaminants cannot be removed from the single-pass RO system. Frequently, the boron rejection level in the single-pass RO process at normal pH (7–8) of seawater was found to be between 40 to 78% [7,8,9,10] and around 30% for brackish water [4,11,12]. That might be credited to the small molecular size of un-dissociated boric acid (H_3_BO_3_) at the normal condition of seawater. Consequently, boron diffuses through the RO membrane to the permeate side. Recently, the WHO (World Health Organization) announced that the standard concentration of Boron should not surpass 2.4 ppm in freshwater. The European Union and South East Asia have set the maximum boron concentration to 1 ppm, which is difficult and expensive to fulfil via a single-pass RO system, due to the operational limitations. Furthermore, raising the pH value to remove boron can lead to Mg(OH)_2_ (magnesium hydroxide) and Ca(OH)_2_ (calcium hydroxide) precipitation on the membrane surface (scaling phenomenon) [13,14].

Some desalination processes such as the second-pass RO system or hybrid desalination system are designed to remove boron from the permeate product, however, they are insufficient in terms of energy and chemical consumption. Moreover, the most challenging RO problem is the high amount (in volume) of the concentrated brine which has organic and inorganic contaminants. The traditional disposal of concentrated RO brine (about 55,000 ppm) is discharged into seawater or injected into deep wells, which has a negative environmental impact. Furthermore, it is not beneficial to treat the concentrated brine via RO, due to the high energy needed (because of increases in the osmotic pressure) and high probability chance of fouling and scaling formation. Nonetheless, Membrane Distillation (MD) could be utilized to treat the RO brine to increase freshwater production and reduce the RO brine volume. This fact can be attributed to MD being less sensitive to feed salinity than RO [15].

### 1.2. Membrane Distillation (MD)

MD is a promising method for desalination and wastewater treatment purposes with high retention that cannot be achieved by traditional methods [16,17,18,19]. In the MD process, the water vapor only goes through the hydrophobic membrane. MD has various attractive aspects such as a low operating pressure and temperature as compared to conventional desalination technologies. The MD system can be collaborated with other separation technologies to generate an integrated (a unified) continuous and discrete separation system. Moreover, MD has the capability to exploit different energy sources, such as solar energy. Despite membrane pore wetting, MD shows much lower membrane fouling and scaling as compared with Nanofiltration (NF) and RO [20].

Flow rate and feed temperature, along with the membrane type, play a major role in the permeate flux value. The vapor pressure difference caused by the difference in the temperature across the membrane surface is considered a driving force for the MD. Additionally, a dusty gas theory is utilized to portray the mass exchange within the MD process. In this theory, the relationship between mass transfer and molecule collisions and/or molecule collisions with internal membrane surface have been linked [21].

MD membranes are highly subject to fouling and wetting phenomena. The main reasons for membrane wetting are: going beyond the liquid entry pressure (LEP) and membrane fouling. Liquid entry pressure (LEP) is a critical membrane characteristic. LEP is the minimum transmembrane pressure value needed for a feed solution to pass through the large pores (*r_max_*). The LEP value should always be higher than the hydrostatic pressure in order to prevent membrane wetting. LEP can be evaluated by [22,23,24]:

(2) $$\Delta P=P_{F}-P_{P}=\;\frac{-2B\gamma_{l}\;cos\theta }{r_{max}}.\;$$

where $\gamma_{l},\theta \;and\;r_{max}$ are the liquid surface tension, contact angle, and maximum pore size, respectively. It was proposed to use poly(vinyl alcohol) (PVA) as a coating material [25] or a cover for the membrane surface by surface micropillars [26] to resolve these issues.

Many different popular MD configurations can be utilized to treat saline or contaminated solutions such as direct contact (DCMD), vacuum (VMD), and air gap (AGMD). In the DCMD configuration, the hot solution and the cooling water are in direct contact with the membrane’s surfaces. The heat loss is one of the drawbacks to this configuration. In order to resolve this issue, non-circulating air was introduced between the cooling fluid and membrane surface (AGMD configuration) [21]. The permeate flux in AGMD is less than DCMD, due to mass transfer resistance. In order to obtain a higher permeate flux, the gap between the condensation surface and the membrane has to be filled up with distilled water, resulting in a reduced mass transfer resistance. This type of configuration is called Permeate Gap membrane distillation (PGMD).

To the authors’ latest knowledge, there are very limited accessible studies dealing with boron removal from seawater via the MD process. For instance, DCMD was performed to remove boron from seawater by the PVDF membrane [27]. They concluded that the DCMD was efficient for seawater desalination, due to the stability of the permeate flux and high boron rejection. Boubakri et al. [28] explored the impact of operating parameters on boron removal by DCMD. It was found that DCMD could produce high water quality at high boron concentration. Additionally, they noticed that the retention of boron at a low pH was high (90%) and stable. Likewise, Hou et al. [29] reported that the boron rejection relied less on the feed pH and salt concentration.

A comparative study was done between permeate gap membrane distillation (PGMD) and air gap membrane distillation (AGMD) [30] in treating aqueous salt solutions. The permeate flux was enhanced significantly when permeate gap membrane distillation was used.

The RO process has shown insufficient boron removal at a moderate level of pH, due to boric acid presence which can disperse through the RO membrane easily [28,31]. There have not been any studies available, to the best of the authors’ knowledge, dealing with boron removal for the permeate of the single-pass RO system via membrane distillation (MD). This research examines the practicality application of MD over a wide range of boron concentrations. A comparative study is conducted to evaluate the best MD technologies based on boron retention at different operating parameters. In this experimental work, AGMD, PGMD, and VMD are employed to assess the feasibility of MD as one of the alternative methods to the single-pass RO for effective boron removal. Furthermore, AGMD, PGMD, and VMD are implemented to treat synthetic seawater (Arabian Gulf concentration) to test and assess their effectiveness.

## 2. Experimental Procedure and Material

### 2.1. Equipment and Materials

Boric acid (H_3_BO_3_) was used to prepare different boron concentrations, which were 1.5, 7 and 30 ppm. Those concentrations of boron were selected on the basis of the concentration of boron in the permeate side of the single-pass RO system [3,14], Arabian Gulf [3], and contaminated brackish water [4,14]. Moreover, NaCl, Na_2_SO_4_, MgCl_2_, CaCl_2_, and KCl were used to prepare synthetic seawater (Sigma-Aldrich, St. Louis, MO, USA). The PTFE membrane (0.2 µm pore size) was commercially obtained from Sterlitech Corporation (Kent, WA, USA).

**Figure 1:**
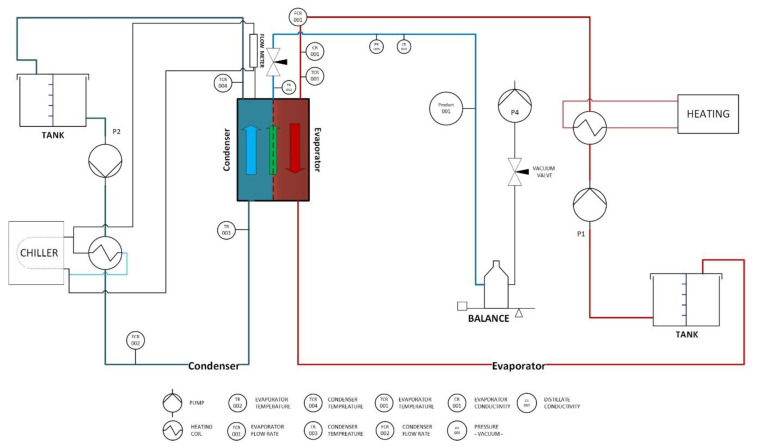
Illustrative diagram of the laboratory Membrane Distillation (MD) system.

AGMD, PGMD, and VMD configurations were implemented to treat an aqueous boron solution, as shown in Figure 1. The impact of a wide range of pH values (3, 9, and 11) on boron retention was examined too. Furthermore, synthetic seawater of the Arabian Gulf concentration (which contained 7 ppm of boron) was treated by AGMD, PGMD, and VMD to measure the performance (permeate flux and rejection factor) of these modules in the presence of different salt components. The composition of the seawater was prepared in the laboratory, as demonstrated in Table 1.

Polytetrafluoroethylene (PTFE) membrane in flat sheet form is utilized to fulfill this study. The PTFE membrane is considered a microporous hydrophobic membrane. The main features of the PTFE membrane are listed in Table 2. It is noteworthy to mention that PTFE has an excellent chemical resistance and is thermally stable to high temperatures.

**Figure 2:**
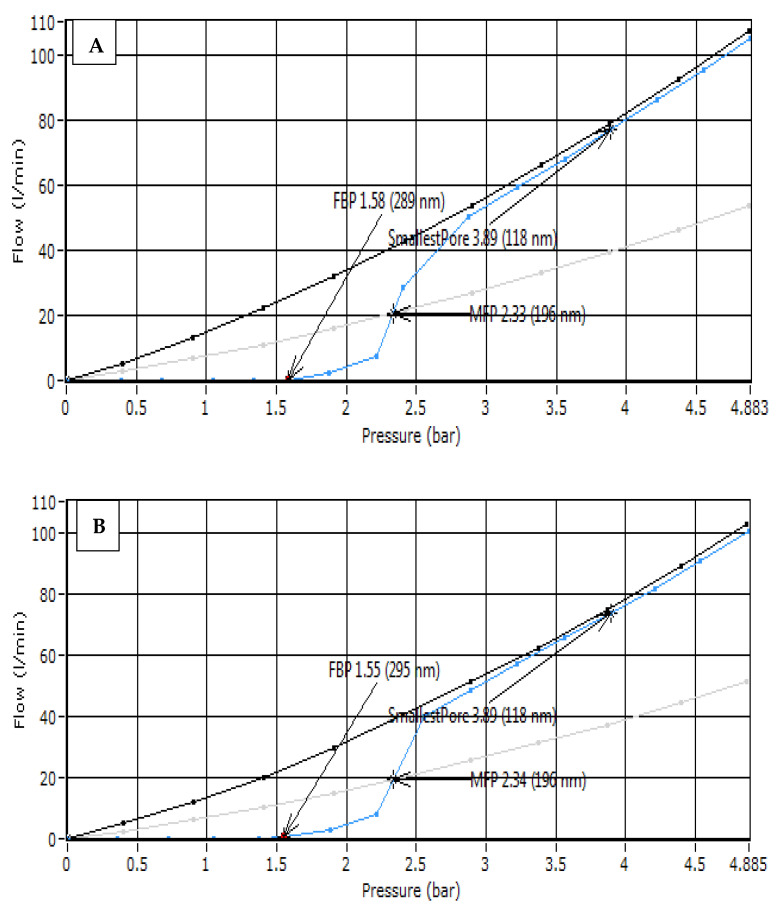
Capillary Flow Porometry (bubble point) method for: (**A**) new polytetrafluoroethylene (PTFE) membrane, (**B**) used PTFE membrane at feed temperature = 70 °C and initial boron concentrations 30 ppm.

The capillary flow porometry (bubble point) method was used to measure the membrane pore size for the new and used PTFE membranes. As shown in Figure 2, the mean pore size for the new and used PTFE membranes are the same (0.196 µm). As a result, the membranes were thermally stable.

### 2.2. Experimental Procedure and Operating Parameters

AGMD, PGMD, and VMD modules were applied to treat aqueous boron solutions and synthetic seawater. The plate and frame module which consists of three separate sections was used in this work. The right side section was filled by the hot solution and the left side was filled by a coolant liquid. The water vapor diffuses through the membrane and after that collects within the middle section (permeation section), which is around 4 mm.

The impact of the operating conditions such as the feed temperature and feed circulation (feed flow) rate on the rejection factor was inspected. For example, the impact of the boron solutions at different circulation rates (0.9, 3, and 5 L/min) was examined. The feed circulation can be controlled by altering the pump speed to attain the specified rate at a fixed temperature. The aqueous solution was heated up to 50 °C and sent to the right section of the membrane module. The feed temperature impact (40, 50, 60, and 70 °C) on the boron retention was analyzed. The feed temperature (at invariable flow rate) was monitored to the specified temperature. Additionally, the cooling liquid flow rate and temperature were kept constant during the experiment.

It is worth mentioning that the temperatures of coolant fluid, feed solution, and the electrical conductivity for the feed and permeate were continuously measured and recorded.

Flux (*J*) for 5 h was estimated by measuring the weight of the collected pure water (permeate):

(3) $$J=\;\frac{W}{A\;\Delta t}.\;$$

where *A* is the effective area of membrane and *W* is the obtained permeate weight.

It is worthwhile that the experimental tests were achieved by various MD configurations with a membrane effective area of 0.006 m^2^. Each experiment was conducted for 5 h and repeated two times for AGMD, PGMD, and VMD respectively. The average reading was computed and considered. For each MD configuration, the effect of feed flow rate on the boron removal was tested first, and then the impact of temperature was tested. To insure that the membrane structure did not change, the experiments that study the impact of the feed temperature at 70 °C were implemented at the end of each MD configuration. The membrane was replaced in two cases: either by the wetting incidence in the membrane pores (boron rejection less than 98.5%) or by the changing of the MD configuration.

In addition, Inductive Couple Plasma (ICP (PerkinElmer, Waltham, MA, USA)) and Atomic Absorption Spectrometry (AAS (PerkinElmer, Waltham, MA, USA)) were employed to measure the boron concentration in the feed (C_f_,) and permeate (C_p_). It is worth mentioning that standard boron solutions were made to measure the accuracy of ICP and AAS. The rejection factor can be calculated;

(4) $$\mathrm{Rejection}\;\mathrm{Factor}=\left(1-\frac{C_{p}}{C_{f}}\right)\times 100$$

## 3. Results and Discussion

Various concentrations of boron at different operating conditions were tested to evaluate the AGMD, PGMD, and VMD performances. In addition, the impact of the pH solution on the rejection factor was inspected.

### 3.1. Influence of Circulation Rate

In order to examine the influence of the circulation rate and boron concentration on the AGMD, PGMD, and VMD performance at an invariable temperatures for feed and cooling fluids, several lab experiment were carried out at various circulation rates (0.9, 3, and 5 L/min) and initial boron concentrations (1.5, 7, and 30 ppm). Boron concentrations 1.5, 7, and 30 ppm represent the boron concentration in the single-pass RO, Arabian Gulf, and contaminated brackish water, respectively.

**Figure 3:**
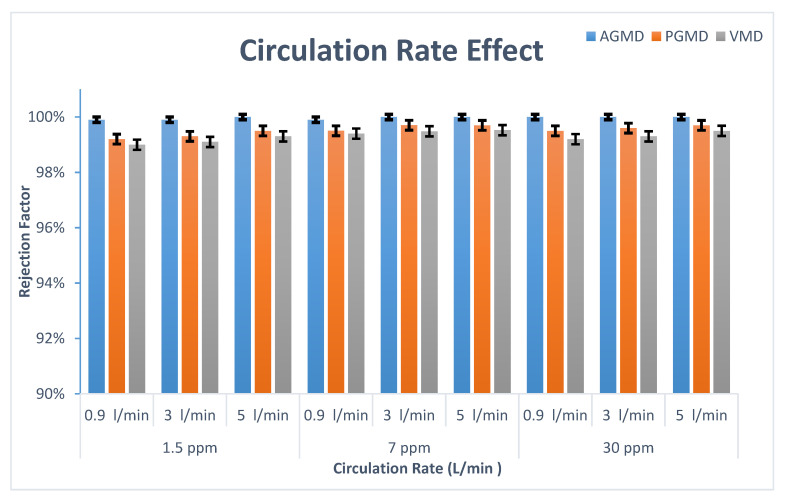
Impact of feed circulation rate and initial boron concentration on boron rejection for Air Gap Membrane Distillation (AGMD), Permeate Gap Membrane Distillation (PGMD), and Vacuum Membrane Distillation (VMD).

Figure 3 demonstrates the variation of boron retention as the circulation rate and initial boron concentration change. It can be concluded from the figure that MD configurations can reject boron over a wide range of boron concentrations and circulation rates. For example, the boron concentrations in permeate flux of PGMD and VMD at 5 L/min and 1.5 ppm (initial boron concentration) were 0.005 and 0.007 ppm, respectively. AGMD, as shown in Figure 3, can almost reject boron completely at different circulation rates, whereas the boron rejection performance for PGMD and VMD varied from 99% to 99.5%. This result can be attributed to the membrane penetration incidence in the large pores. The feed solution passed through the large hydrophobic membrane pores to the permeate side. Boric acid has a small molecular size that might penetrate through the wetted pores to the permeate side (diffusion). It is noteworthy that the risk of membrane wetting in the AGMD module is lower than that of other MD modules. Therefore, AGMD achieved better removal efficiency.

High rejection performance for boron at Arabian Gulf concentration (7 ppm) was proven by AGMD, PGMD, and VMD at different flow rates. The rejection factor differed from 99.4–100%.

It was also observed that the membrane retention for boron improved when the circulation rate rose, notwithstanding the concentration. For instance, boron retention at an initial concentration of 30 ppm for VMD was 99.2%, 99.3%, and 99.5% at 0.9, 3, and 5 L/min, respectively. Concurrently, a higher flux occurred at higher feed circulation rates. When the feed circulation rate increased from 0.9 L/min, 3 L/min, and 5 L/min, the permeate flux for VMD was 6.9, 7.4, and 7.7 kg/m^2^h, respectively. This can be interpreted by the increase of the feed circulation rate which enhances the mass transfer coefficient, leading to a rise in the permeate flux. Furthermore, the impact of the increasing boron concentration was minimal on the MD performance, particularly in AGMD, followed by PGMD and VMD.

**Figure 4:**
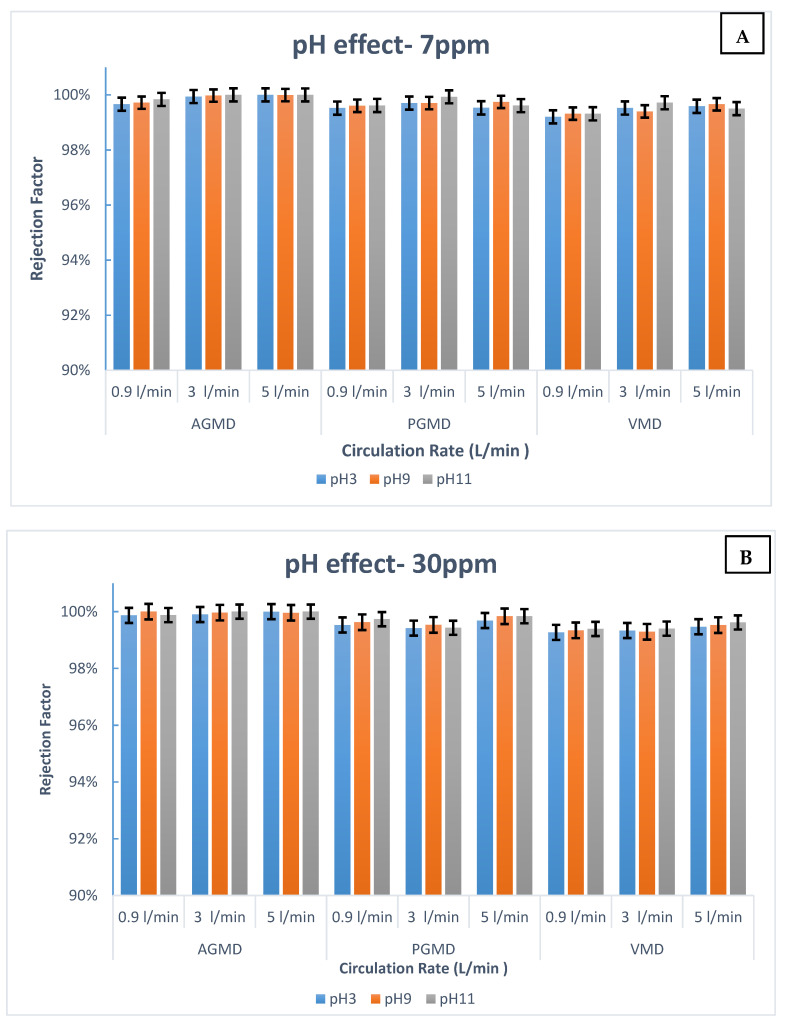
Influence of pH on boron retention at different feed circulation rates and initial boron concentrations: (**A**) 7 ppm and (**B**) 30 ppm for AGMD, PGMD, and VMD.

Because of the conventional membrane technologies that are immensely dependent on the pH feed solution, the impact of pH on the boron rejection performance for AGMD, PGMD, and VMD was tested at diverse circulation rates and pH values. Experiments were conducted with pH values: 3, 9, and 11. The initial boron concentrations were 7 ppm and 30 ppm. The circulation rate varied from 0.9 to 5 L/min at invariable temperature. Figure 4 demonstrates the variation of the boron rejection factor as a function of the pH and circulation rate. As evident from Figure 4, the removal of boron was almost steady and kept at a satisfactory level between 99.2–100% in the whole filtration process, which implies that high acidity has no notable effect on the AGMD, PGMD, or VMD performance. A similar finding for the DCMD module was reported by Boubakri et al. [28] and Hou et al. [29].

Figure 4A shows that the boron rejection was higher than 99.2% in all feed pH ranges. The boron concentration in the water product was less than 0.007 ppm, even at a feed of boron concentration as high as 7 ppm. Additionally in Figure 4, it can be seen that AGMD shows excellent performance regardless of the value of the pH. As exhibited in Figure 4B, it is important to note that, the rejection factor at a high boron concentration for PGMD and VMD was almost stable at 99.4% when decreasing the feed pH up to 3. The driving force in the MD process is the vapor pressure difference across the membrane’s surface, which is less dependent on the pH value of the feed. Therefore, boron removal can be achieved at a high acidity feed solution. As a result, AGMD, PGMD, and VMD are independent of the boric acid/borate ion concentration. A similar finding has been observed [28,29,33]. Therefore, these modules can be considered as alternative technologies which then can be used to stand alone or, while connected to other technologies, to eliminate boron from saline solutions at a natural pH value.

### 3.2. Impact of Feed Temperature

Boron removal at various feed temperatures by the AGMD, PGMD, and VMD configurations was examined at a constant coolant temperature and feed circulation rate.

**Figure 5:**
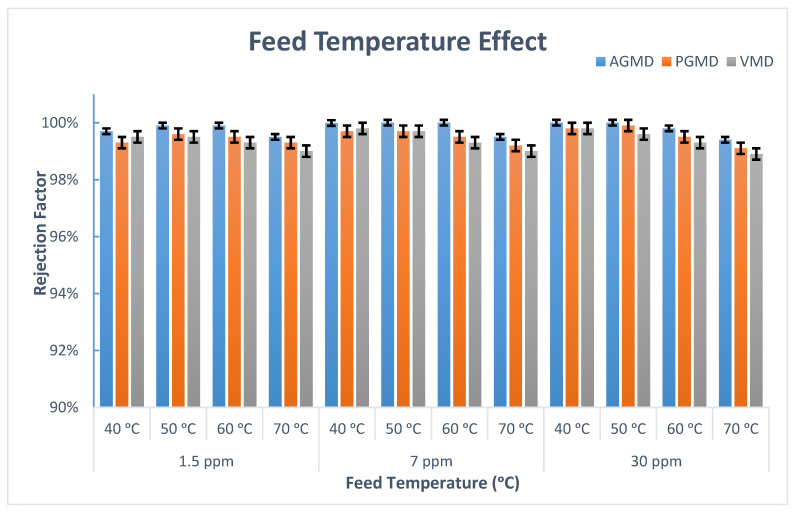
Boron rejection for AGMD, PGMD, and VMD at different feed temperatures and initial boron concentrations.

As revealed in Figure 5, the boron removal for MD configurations was excellent and steady over a vast concentration and temperature range. For example, the boron removal for AGMD was almost 100% over 40–60 °C. The findings reveal that the impact of feed temperature on boron removal was very limited. Additionally, as the feed temperature increased from 40 to 70 °C, the permeate flux for AGMD increased by 37%. As regards the eligibility of the PGMD and VMD for boron rejection, both show resemblance in boron removal efficiency (about a 99% removal) over the same temperature range. The boron concentration in the permeate side for AGMD, PGMD, and VMD was lower than 0.01 ppm, which indicated that the membrane wetting had not occurred during the experiment.

**Figure 6:**
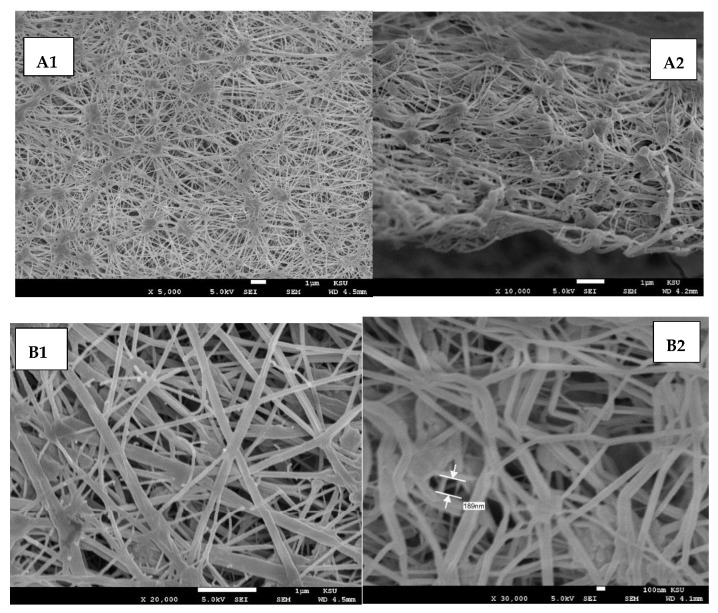
SEM images for PTFE membrane: (**A1**) surface image for new membrane, (**A2**) cross section image for new membrane, (**B1**) surface image for used membrane, and (**B2**) cross section image for used membrane at feed temperature = 70 °C and initial boron concentrations at 30 ppm.

However, it was noticed from Figure 5 that the boron retention for MD modules had slightly decreased at 70 °C compared to the lower temperatures, which might be credited to the membrane penetration incidence for the large pores. The feed solution passed through the wetted hydrophobic membrane pores to the permeate side (diffusion). Saffarini et al. [34] stated that the liquid entry pressure (LEP) value is affected negatively by the reduced feed surface tension, membrane hydrophobicity, and increased feed temperature. Moreover, the PTFE membrane was thermally stable and the membrane’s structure was unchanged (e.g., membrane pores), as is shown in Figure 6.

**Figure 7:**
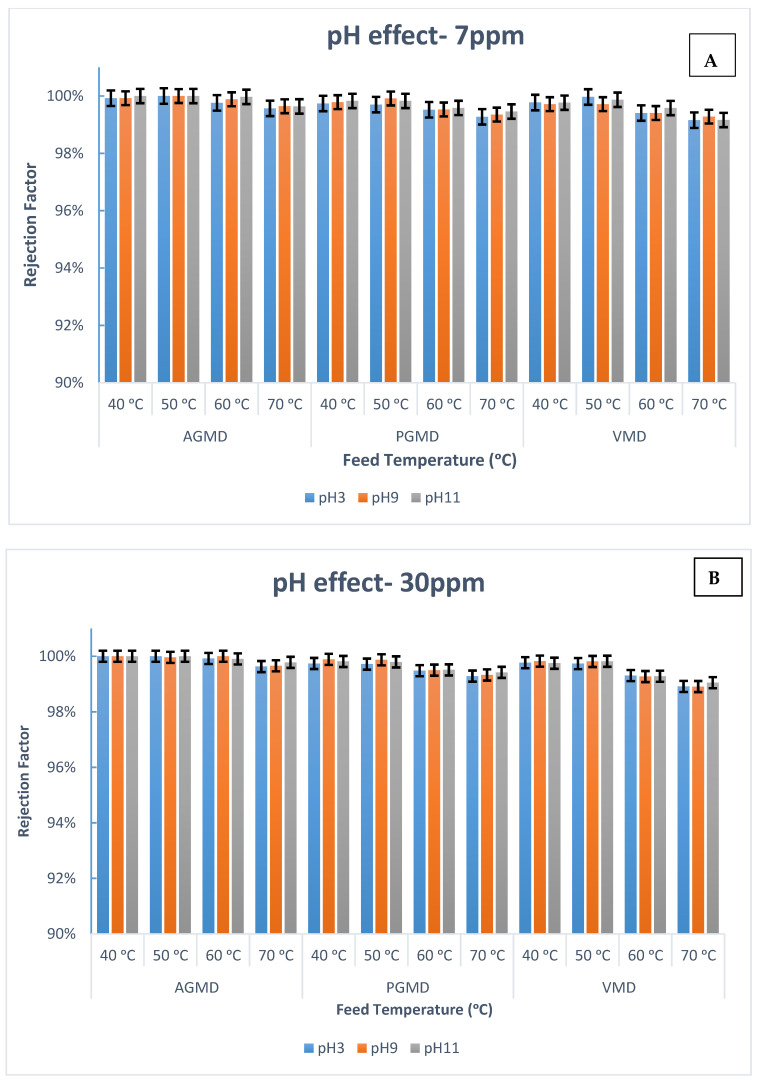
Boron rejection for AGMD, PGMD, and VMD at different pH values and feed temperatures for initial boron concentrations: (**A**) 7 ppm; (**B**) 30 ppm.

To assess the impact of feed pH on the filtration performance for the AGMD, PGMD, and VMD processes, a number of lab experiments were conducted with the pH range of 3–11. The effects of boron removal by AGMD, PGMD, and VMD configurations at various pH values are presented in Figure 7. The boron removal efficiencies for all MD modules were almost within 99% to 100%, indicating the minimum effect of the pH value on the AGMD, PGMD, and VMD performances. This is likely because MD is independent of the boric acid/borate ion concentration. The risk of membrane wetting in MD modules is low, especially at moderate operating parameters. Moreover, the driving force in the MD process is the vapor pressure difference across the membrane surface. Therefore, boron removal relies less on the feed pH.

Restating that the boron retention for MD modules slightly declined at 70 °C compared to the lower temperatures, which might be credited to the membrane penetration incidence. For instance, the boron removal at a low pH for VMD at a 7 ppm initial boron concentration was 99.1%, which indicates 0.01 ppm boron in the permeate side. Consequently, MD technology can be taken into consideration as an alternative technology to eliminate boron from aqueous solutions at high temperature operations.

### 3.3. Synthetic Seawater

Under constant operating conditions such as feed and condensing temperatures, the impact of seawater circulation rate from 0.9 to 5 L/min on the permeate flux and the rejection factor was inspected. In the previous experiments for AGMD, PGMD, and VMD, the feed temperature at 60 °C showed better flux and an excellent rejection factor, therefore, it was selected to be the operating feed temperature.

**Figure 8:**
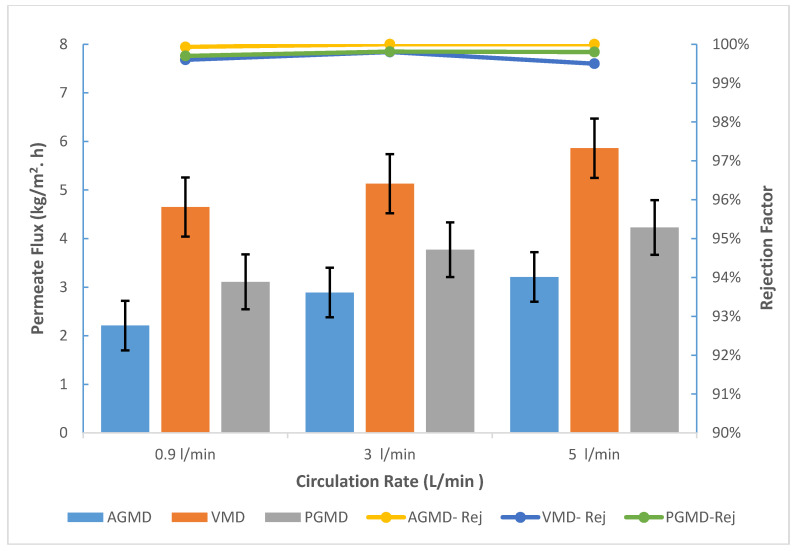
Permeate flux and salt rejection for AGMD, PGMD, and VMD for synthetic seawater.

It is shown in Figure 8 that an increase in the permeate flux is due to an increase in the feed circulation rate. For example, the permeate flux for AGMD, PGMD, and VMD at 3 L/min was 2.8, 3.7, and 5.1 kg/m^2^·h. When the feed circulation rate was increased to 5 L/min, the improvement in the permeate flux increased by 11%, 12%, and 14% for AGMD, PGMD, and VMD, respectively.

This can be elucidated by the temperature and concentration polarization reduction. Raising the circulation flow rate will decrease the concentration difference between the bulk and the membrane surface. Additionally, the temperature polarization effect at the membrane feed side will decrease, due to the rise in feed circulation velocity. As a result, the temperature difference between the membrane surface and the feed bulk will reduce. Thus, the mass transfer and heat transfer coefficients at the feed boundary will increase.

It can be understood from Figure 8 that the boron removal was about 99.5% during the experiments and there was no concealable change over the flowrate changing. Boron concentration in the pure water product was about 0.01 ppm. Additionally, the electrical conductivity of the permeate varied between 2.3–6.1 μs/cm. As a result, the MD configurations were efficient for seawater desalination and boron removal.

Finally, AGMD, PGMD, and VMD are all eligible to treat seawater.

Nonetheless, unlike PGMD and AGMD, VMD showed an increased permeate flux. On the other hand, AGMD showed excellent boron rejection. For this reason, AGMD is proposed to be an alternative solution to the second-pass RO for boron removal. In addition, VMD is proposed to be an alternative technology for seawater desalination.

## 4. Conclusions

A comparative study was conducted at various operating parameters to evaluate the best MD technologies over a wide range of boron concentrations (1.5, 7, and 30 ppm). AGMD, PGMD, and VMD were used to assess the practicality of MD as one of the alternative methods to treat the first-pass permeate RO for effective boron removal. Furthermore, AGMD, PGMD, and VMD were implemented to treat synthetic seawater and evaluate their efficacy for boron removal. Summarized below are the essential findings:

- MD is a worthy technology for boron removal and seawater desalination (which varied between 99.3–100%).
- Boron removal for AGMD was excellent and stable over a wide variety of concentrations, temperature range, and flow rate, while taking note that PGMD and VMD show better permeate fluxes.
- The permeate fluxes of AGMD, PGMD, and VMD for synthetic seawater at 60 °C and 5 L/min were 3.21, 4.23, and 5.86 kg/m^2^·h. Additionally, the boron concentration was about 0.01 ppm.
- When the feed circulation rate increased, the boron rejection for AGMD, PGMD, and VMD increased no matter the concentration.
- For better boron removal by MD, it is recommended not to exceed 70 °C in the feed side to prevent membrane wetting.
- AGMD is proposed to be an alternative solution to the second-pass RO for boron removal. In addition, VMD is proposed to be an alternative technology for seawater desalination
- There is no noteworthy impact on boron removal from the high acidity of the feed solution (PH value), which can be considered an attractive feature. Whereas the traditional membrane filtration depends highly on the pH feed solution.

## Figures and Tables

**Table 1:** The main components of synthetic seawater that were used in this study [32].

Salt Component	Concentration (ppm)
Chloride, Cl^−^	23,000
Sodium, Na^+^	15,850
Sulfate, SO_4_^2−^	3200
Magnesium, Mg^2+^	1765
Calcium, Ca^2+^	500
Potassium, K^+^	460
Boron, B	7
pH	7.3
Total dissolved solids, TDS	45,000

**Table 2:** Membrane features utilized in the experimental study, as specified by the producer.

Parameter	Specification
Material	PTFE
Commercial name	TF200
Mean pore size	0.2 µm
Liquid entry pressure (LEP)	2.55 bar
Thickness	175 µm
Membrane support	Polypropylene
Manufacturer	Sterlitech corporation
